# Intrapleural Tenecteplase for Complicated Parapneumonic Pleural Effusion

**DOI:** 10.1155/2021/2206692

**Published:** 2021-10-08

**Authors:** Husain Kadhem, Kannan Sridharan, Naser Naser

**Affiliations:** ^1^Department of Internal Medicine, Salmaniya Medical Complex, Manama, Bahrain; ^2^Department of Pharmacology & Therapeutics, College of Medicine & Medical Sciences, Arabian Gulf University, Manama, Bahrain

## Abstract

Intrapleural thrombolytics have shown promising results in complicated parapneumonic pleural effusions reducing the need for surgical interventions. Until now, studies have evaluated primarily streptokinase, urokinase, and recently, alteplase. In this case series, we share our experience with tenecteplase—a tissue-specific plasminogen activator in 15 patients. We observed that tenecteplase is effective in 14/15 (93.3%) of our patients, and none of them had any bleeding episodes.

## 1. Introduction

Parapneumonic pleural effusion occurs in approximately 35-60% of patients with pneumonia [1]. Conventional management practice of chest tube drainage and antimicrobial drugs in complicated parapneumonic effusion is effective in three-fourths of the cases [2]. Surgical management through pulmonary decortication was observed with an overall mortality of 3% and postoperative complications in 39% of the patients [3]. Pleural infections predispose to increased deposition of plasminogen activator inhibitors 1 and 2, thereby increasing the intrapleural fibrin deposition resulting in loculations [4]. Intrapleural thrombolytics facilitate pleural fluid drainage in complicated and loculated effusions by breaking up the loculations. A Cochrane review that included studies with streptokinase, urokinase, and alteplase concluded a significantly reduced mortality and requirement of surgical intervention [5]. Until date, there is only one preliminary report available with the use of tenecteplase as intrapleural thrombolytic agent [6]. Tenecteplase, like alteplase, is a recombinant tissue plasminogen activator selective to tissue plasminogen, thus expected with lower incidences of adverse events such as bleeding, following the systemic administration [7]. We wish to share our experience in using tenecteplase for facilitating the pleural fluid drainage in 15 patients with parapneumonic effusions in the present case series.

## 2. Case Series

We encountered 15 patients with parapneumonic effusion with the median (range) age of 43 (33-72) years and except for one; all the study participants were males. Written consent was obtained from each study participant. The baseline characteristics of the study participants are listed in Table 1. All the included patients had exudative polymorphic pleural fluid on aspiration. Most of the study participants had shown elevated total white blood cell count and C-reactive protein levels. Three participants were detected with loculated pleural effusion in ultrasonogram while seven by computed tomography. Twelve patients had pleural catheter, while three had chest tube for draining the pleural fluid.

## 3. Description of the Interventions

Following the confirmation of pleural effusion, ultrasound was carried out to identify the biggest pocket for inserting the catheter under ultrasound guidance and with local anesthesia. Special pleural catheter was prepared by keeping the stylet inside the catheter. After administration of local anesthetics, the needle connected to a 20 cc syringe was inserted until the pleural fluid drained freely. Then, the syringe was disconnected, the guide wire was introduced, and after removing the needle, a space was created by blade to insert the dilator to open the tract for the catheter. After dilator removal, a catheter was introduced inside pleural cavity. As soon as the catheter was inside the pleural cavity, the stylet and guide wire were removed; catheter was opened and connected to a drainage box. Suction (ranging from 15 to 20 mmHg) was applied. Thoracic tube insertion was carried out in the traditional way.

Tenecteplase solution was prepared by keeping 3000 IU tenecteplase with 40 cc of 0.9 normal saline mixed 10 cc of 1% of lidocaine. The whole mixture was kept in a 50 mL syringe. The syringe was then connected to a 3-way lock catheter, and the solution was pushed inside the catheter followed by 20 mL normal saline flushing. The catheter was closed for one hour and then drained.

## 4. Details regarding Tenecteplase Administration and Postadministration Events

All the study participants were administered tenecteplase at 3000 IU. The key details of the intervention and postinterventional events are listed in Table 2. Following tenecteplase, all the participants had complete/near complete drainage of pleural fluid except one who needed additional thoracotomy with decortication for complete resolution. Ten (66.7%) had complete resolution of chest X-ray findings following the drainage after tenecteplase while four (26.7%) showed significant improvement from the baseline. Nearly half (8, 53.3%) required analgesics for pain control following the drainage. The administration of tenecteplase was considered to have failed when there was no improvement in the chest X-ray or absence of any pleural fluid being drained or persistence of fever and/or chest pain. The interval time for evaluation and readministration of tenecteplase was 24 hours.

## 5. Discussion

We observed that tenecteplase is effective and safe in our patients with complicated parapneumonic effusion. The findings were like the only other report with tenecteplase where the authors have observed an efficacy rate of 92.1% (35/38 patients) and adverse events in only 6.9% (4/38) patients [6]. We observed the treatment failure rate of only one (out of the total 15) patient. Studies have reported around 27% failure rate with chest tube drainage alone [8]. Deoxyribonuclease (DNase) significantly decreases the viscosity of the pus in the pleural fluid and eases the drainage [9]. Intrapleural alteplase with DNase was observed to be successful in 58/61 (93.4%) of patients with bleeding in the pleural fluid requiring blood transfusion encountered in three (4.9%) patients [10]. Thrombolytics alone were observed with significantly lower success rate in large randomized clinical trials [11]. Tenecteplase with DNase is yet to be trialled, and it would be interesting and useful to evaluate the outcomes with this combination. Similarly, tenecteplase was observed to be effective even in our patients with 3 locules as detected by CT scan. Tenecteplase is a long-acting thrombolytic drug thus requiring fewer doses of administration for prolonged effect. We observed that except for two patients (due to partial response), the remaining required only one- or two-times administration. The prolonged duration of action of tenecteplase may be an advantage in patients with pleural effusion associated with inoperable lung cancer. We did not observe any bleeding episodes with tenecteplase despite two patients with the history of cerebro-vascular accident. Fibrin-specific thrombolytic has been shown to be effective and safe even when used in patients with thalassemia with baseline anaemia and on anticoagulation therapy [12]. The only side effect observed following intrapleural administration was pain at the local site that was effectively ameliorated in all the patients with analgesics. In fact, nearly half of our patients (8/15) did not require any analgesic as the pain was self-limiting.

## 6. Conclusion

We observed that intrapleural tenecteplase is effective and safe for facilitating drainage of pleural fluid in parapneumonic effusions. High quality randomized clinical trials with/without DNase combination are needed for confirming the therapeutic benefit of intrapleural tenecteplase.

## Figures and Tables

**Table 1:** Key characteristics of the study participants.

S. No.	Age; sex	Concomitant disorder	Baseline respiratory symptoms	Baseline total WBC count (count × 109/L)	Baseline CRP (mg/L)	Aspirated pleural fluid glucose (mmol/L)	Aspirated pleural fluid WBC count (cells/*μ*L)	Neutrophil percentage in the pleural fluid (%)	Number of loculations as detected in USG	Number of loculations as detected in CT
1	41; M	None	F, P, D	11	305	Not available	790	82	2	3
2	54; M	Dyslipidemia	F, P, C, S	8	161	6.9	7890	70	1	2
3	33; M	None	P, C, S	16	241	6.8	1420	30	1	1
4	37; M	COVID-19	F, P, D	15	160	Not available	2100	50	1	Not done
5	37; M	None	F, P	14	Not available	2.6	8740	79	1	1
6	34; M	None	F, P, D	24	191	0.2	1306	NA	1	2
7	49; M	Diabetes	P	13	160	7.3	830	87	1	Not done
8	72; M	Liver transplantation and CVA	C, S, D	12	Not available	Not available	1610	80	1	2
9	40; Fe	None	F, P, D	13	Not available	1.1	21780	84	1	1
10	41; M	G6PD deficiency	F, P, D	11	180	0.2	2180	83	1	1
11	56; M	G6PD deficiency, diabetes	F, P, C, S, D	8.8	291	3.9	890	89	2	3
12	48; M	Diabetes, systemic hypertension	P, C, D	13.3	202	13.1	1580	65	1	2
13	68; M	Diabetes, IHD, atrial fibrillation	D	14	206	7.7	2160	88	1	1
14	71; M	Dyslipidemia, CVA	F, P, C, S, D	10	Not available	0.2	7210	90	1	1
15	64; M	None	D	16	Not available	2.2	520	61	2	3

M: male; Fe: female; COVID-19: coronavirus 2019 infection; CVA: cerebrovascular accident; IHD: ischemic heart disease; F: fever; P: chest pain; C: cough; D: dyspnea; USG: ultrasonogram; CT: computed tomography.

**Table 2:** Details regarding the intervention (tenecteplase) and postinterventional events.

S. No.	Frequency of tenecteplase application	Additional interventions needed for pleural drainage after tenecteplase	Chest X-ray picture following tenecteplase administration	Concomitant analgesics
1	Four	None	Improved	Paracetamol and naproxen
2	Once	None	Clear	Paracetamol and ibuprofen
3	Once	None	Clear	None
4	Twice	None	Improved	None
5	Twice	None	Clear	Tramadol
6	Twice	Thoracotomy and decortication	No improvement	None
7	Twice	None	Clear	Tramadol
8	Twice	None	Clear	None
9	Twice	None	Clear	Tramadol
10	Twice	None	Clear	None
11	Once	None	Clear	Tramadol
12	Twice	None	Improved	Tramadol
13	Twice	None	Clear	Paracetamol
14	Once	None	Improved	None
15	Four	None	Clear	None

## Additional Points

*Key Messages*. (i) Intrapleural thrombolytics are effective in facilitating the drainage of complex pleural effusions. (ii) Previous studies have used streptokinase, urokinase, and alteplase. Only one published study is available encountering tenecteplase. (iii) We observed that tenecteplase is effective and safe in our patients with complicated parapneumonic pleural effusions.
